# Impact of Preoperative Halo Traction on Cobb Angle Reduction in Adolescent Idiopathic Scoliosis: A Retrospective Analysis

**DOI:** 10.3390/children12081045

**Published:** 2025-08-09

**Authors:** Mihai Bogdan Popescu, Harun Marie, Alexandru Ulici, Sebastian Nicolae Ionescu, Adelina Ionescu, Ioana Alexandra Popescu, Alexandru Herdea

**Affiliations:** 1 11th Department of Pediatric Orthopedics, Carol Davila University of Medicine and Pharmacy, Bd. Eroii Sanitari Nr. 8, 050474 Bucharest, Romania; mihai-bogdan.popescu@drd.umfcd.ro (M.B.P.); alexandru.ulici@umfcd.ro (A.U.); adelina_ionescu@spitalulgrigorealexandrescu.ro (A.I.); alexandru.herdea@umfcd.ro (A.H.); 2 Pediatric Orthopedics Department, Grigore Alexandrescu Children’s Emergency Hospital, 011743 Bucharest, Romania; harun_marie@spitalulgrigorealexandrescu.ro (H.M.); ioana-alexandra.popescu0720@stud.umfcd.ro (I.A.P.); 3 Emergency Hospital for Children Maria Sklodowska Curie, 75534 Bucharest, Romania

**Keywords:** halo-gravity traction, adolescent idiopathic scoliosis, Cobb angle, Risser score, Lenke classification, spinal deformity correction, skeletal maturity, spinal fusion, preoperative traction, scoliosis surgery

## Abstract

**Highlights:**

**What are the main findings?**

- Halo-gravity traction reduced the primary Cobb angle by 26.09% and the secondary curve by 26.94% preoperatively, with a total postoperative correction exceeding 64% in both.
- Patient age, Risser score, and Lenke classification did not significantly influence the degree of correction achieved.

**What is the implication of the main finding?**

- Halo-gravity traction is broadly effective and safe across a range of adolescent idiopathic scoliosis cases, regardless of skeletal maturity or curve type.
- A traction period of approximately 24 days appears optimal, offering significant deformity correction with minimal additional benefit from longer durations.

**Abstract:**

Background/Objectives: Adolescent idiopathic scoliosis (AIS) is a three-dimensional spinal deformity often requiring surgical correction in severe cases. Halo-gravity traction (HGT) is commonly employed preoperatively to enhance spinal flexibility and reduce curve severity. This study aimed to evaluate the effectiveness of HGT in reducing Cobb angles in AIS and to assess how patient age, skeletal maturity (Risser score), and curve type (Lenke classification) influence treatment response. Methods: We conducted a retrospective cohort study of 28 AIS patients with Cobb angles > 65° who underwent preoperative HGT followed by posterior spinal fusion. Traction was applied for a mean of 24.64 days, reaching 40–50% of each patient’s body weight. Radiographic measurements were collected pre-traction, post-traction, and postoperatively. Statistical analyses included paired *t*-tests, Pearson correlations, Kruskal–Wallis tests, and linear regression. Results: Mean primary Cobb angle was reduced from 82.46° pre-traction to 61.00° post-traction (26.09% reduction) and further to 29.54° postoperatively (64.58% total reduction). Similar reductions were observed in secondary curves. No statistically significant correlations were found between age or Risser score and the magnitude of correction. Lenke type 3 showed the highest traction response, while type 5 had the greatest surgical gain. Curve type and skeletal maturity did not significantly affect final outcomes. Conclusions: Halo-gravity traction is a safe and effective adjunct in the surgical treatment of severe AIS, achieving substantial Cobb angle reduction. The degree of correction was not significantly influenced by age, Risser score, or curve type, supporting the broad applicability of HGT across adolescent patients.

## 1. Introduction

Scoliosis, a three-dimensional deformity of the spine characterized by lateral curvature and vertebral rotation, is a significant orthopedic condition affecting approximately 0.47–5.2% of the global population, with adolescent idiopathic scoliosis (AIS) being the most common form [1]. Epidemiological studies indicate that the female-to-male ratio in adolescent idiopathic scoliosis increases with age, ranging from 1.5:1 to 3:1 overall and escalating notably with curve severity. Specifically, girls outnumber boys 1.4:1 at Cobb angles of 10–20°, rising to approximately 7.2:1 for curves exceeding 40° [1].

Scoliosis severity is most commonly quantified using the Cobb angle, a radiographic metric defined as the angle between lines drawn along the superior endplate of the uppermost tilted vertebra and the inferior endplate of the lowest tilted vertebra in a coronal (standing PA) X-ray. A Cobb angle ≥ 10° is diagnostic of scoliosis, with ranges further stratified into mild (10–20°), moderate (20–40°), and severe (>40°) curves. Treatment decisions—spanning observation, physical therapy, bracing, or surgical intervention—are guided primarily by this angular measurement and the patient’s skeletal maturity and progression risk [2]. In cases of severe scoliosis, where the Cobb angle exceeds 70°, complementary interventions such as halo traction have gained recognition as a means of reducing curvature and improving spinal flexibility prior to definitive surgical correction [3].

Recent analyses indicate that although the cost of spinal deformity surgery has continued to escalate, the adoption of modern spinal implants—such as pedicle screw and rod constructs—has enhanced corrective precision, fusion stability, and overall patient satisfaction [4,5].

The optimal timing for surgical intervention in idiopathic scoliosis remains a subject of ongoing debate, balancing the risks of early surgery against the potential complications of curve progression if delayed. Early surgical treatment, particularly before skeletal maturity, may allow for better correction and reduce the risk of further deformity and associated cardiopulmonary compromise in severe cases [6]. However, delaying surgery until after peak growth can minimize the risks of crankshaft phenomenon and reduce the need for extended fusion levels [7,8]. Ultimately, surgical timing should be individualized, considering curve magnitude, skeletal maturity, and patient-specific factors.

Halo traction, a technique that involves the application of gradual axial traction through a halo device attached to the skull, has been shown to reduce Cobb angles, improve pulmonary function, and enhance spinal flexibility in patients with severe scoliosis [9,10] and assist surgical correction [11]. It is a well-established preoperative method that facilitates safer and more effective surgical outcomes by gradually improving spinal flexibility and reducing curve severity. Despite its routine use, the optimal indications and predictors of response to traction remain debated. However, the effectiveness of halo traction can vary significantly depending on factors such as skeletal maturity, curve severity, and patient age.

Assessing skeletal maturity is essential in the management of idiopathic scoliosis, as it helps predict the risk of curve progression and guides the timing of intervention. Several methods are available, including the Tanner–Whitehouse method, the Greulich and Pyle atlas, the Sanders staging system based on hand radiographs, and evaluation of the tri-radiate cartilage. However, the Risser score, which assesses the ossification of the iliac crest apophysis on pelvic radiographs, remains one of the most widely used and clinically practical methods. It offers a reliable correlation with remaining growth potential and has become the standard tool in both clinical practice and research due to its reproducibility and ease of interpretation [12,13]. The Risser score, an evaluation of skeletal maturity based on ossification of the iliac crest, is often used to predict curve progression and guide treatment planning [14,15]. In 1958, Joseph C. Risser introduced a radiographic classification system to assess skeletal maturity by evaluating ossification of the left iliac crest apophysis. Patients progress through stages I–V, beginning with Stage I—defined as ossification of less than 25% of the iliac crest apophysis—and culminating in Stage V, which represents complete ossification and fusion. This transition typically occurs over a 1–3-year interval [16].

Despite its widespread use, there is limited consensus on the optimal application of halo traction, particularly in relation to patient-specific factors such as age and skeletal maturity.

The main objective of this study is to investigate the efficacy of halo traction in the management of severe scoliosis in pediatric patients. We quantified its impact on Cobb angle reduction and evaluated how skeletal maturity (Risser score), patient age, and traction duration influence treatment response.

## 2. Materials and Methods

### 2.1. Study Design

We conducted a retrospective cohort analysis to assess the impact of preoperative halo traction on curve evolution and postoperative outcomes in patients with severe scoliosis. This study received approval from the hospital’s ethics committee on 14 March 2025 and was assigned an identification number 9. Written informed consent was obtained from the parents or legal guardians of all participating children. Data were collected from medical records of patients treated at a single orthopedic center between 2018 and 2024.

### 2.2. Participants

This study included patients diagnosed with adolescent idiopathic scoliosis, defined by a Cobb angle greater than 65°, who underwent preoperative halo traction followed by surgical correction (posterior spinal fusion). Patients with congenital, neuromuscular, or post-traumatic scoliosis were excluded, as were those with incomplete medical records or missing key data points.

### 2.3. Study Procedure

Preoperative halo traction was applied using a halo ring attached under general anesthesia to the patient’s skull with 4 titanium pins. The halo was connected to weights through a pulley system in order to apply gradual axial traction. Patients started with no weight added to the system, and afterwards, adding 1 kg of weight per day, the total traction weight reached was 40–50% of each patient’s body weight. Traction was transferable between the bed and a walker or wheelchair. During this period, patients were monitored daily for complications, such as pin site infections or neurological deficits, and were also instructed to notify medical personnel if such complications occurred. Adjustments to the traction weight were made based on patient tolerance and radiographic progress. Following halo traction, patients underwent posterior spinal fusion with instrumentation. All patients underwent posterior instrumented spinal fusion following completion of preoperative halo traction. Fusion levels were selected based on curve type and flexibility, aiming to achieve maximal correction while preserving motion segments. Ponte osteotomies were performed when necessary to increase flexibility and correct thoracic kyphosis.

**Figure 1:**
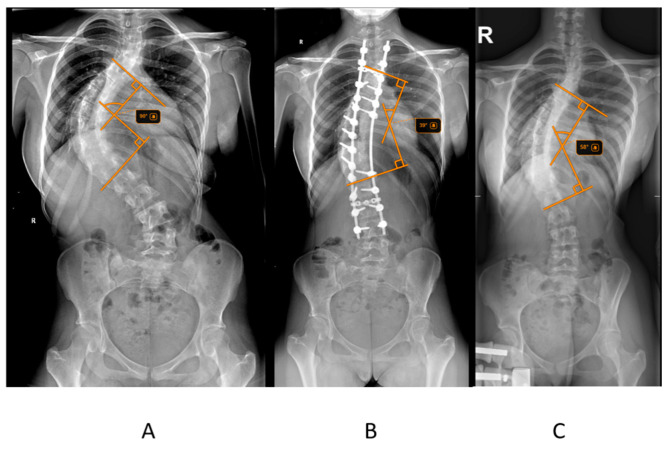
Radiological assessment of a patient from the study group diagnosed with adolescent idiopathic scoliosis. (**A**) Standing anteroposterior (AP) radiograph of the spine taken prior to the initiation of traction, showing the baseline curvature. (**B**) Standing AP radiograph after halo-gravity traction, demonstrating partial correction and increased spinal alignment. (**C**) Standing postoperative AP radiograph illustrating the final spinal alignment following surgical correction and instrumentation.

The primary outcome measured was postoperative Cobb angle improvement, calculated as the difference between the initial Cobb angle and the postoperative Cobb angle (Figure 1). The percentage reduction in Cobb angle after halo traction was also assessed. Radiographic measurements, including the Cobb angle and Risser score, were obtained from pre-traction, post-traction, and postoperative radiographs. The Risser score, which assesses skeletal maturity based on the ossification of the iliac crest, was determined from pelvic radiographs. Radiographic measurements, including the Cobb angle and Risser score, were obtained from pre-traction, post-traction, and postoperative radiographs. To ensure reliability, all Cobb angle measurements were independently performed by two orthopedic observers, and in cases of discrepancy greater than 5°, the average of the two measurements was used for analysis.

### 2.4. Statistical Analysis

Demographic and clinical characteristics, including age, sex, weight, traction weight, and duration, were recorded for each patient.

Statistical analyses were performed using SPSS (version 26.0, IBM Corp., Armonk, NY, USA). Descriptive statistics, including means, standard deviations, and ranges, were calculated for continuous variables such as age, Cobb angles, and Risser scores. A paired *t*-test was used to compare the initial Cobb angle and the postoperative Cobb angle to evaluate the overall improvement in spinal curvature.

To assess the influence of halo traction, the postoperative Cobb angle improvement was analyzed in the context of pre-traction and post-traction changes. Pearson’s correlation was used to explore the relationship between patient age, Risser score, and postoperative Cobb angle improvement. Kruskal–Wallis tested Cobb changes across Lenke types. Additionally, linear regression analysis was performed to identify predictors of postoperative improvement, such as Risser score, age, and initial Cobb angle. A *p*-value of less than 0.05 was considered statistically significant.

## 3. Results

A total of 28 patients met the inclusion criteria and were included in the analysis. The cohort consisted of 6 males and 22 females, with an age range of 12 to 17 years (mean age: 14.86 years). All the patients included in this study underwent preoperative halo-gravity traction for a mean period of 24.64 days of traction followed by surgical correction. The mean Risser score at the time of treatment was 2.75, with a range from 2 to 5, indicating varied skeletal maturity among participants. The mean body weight was 50.5 kg (SD ± 7.56), with values spanning from 39 to 63 kg. Patients underwent halo-gravity traction using an average weight of 19.21 kg (SD ± 2.22), and the mean duration of traction was 24.64 days (SD ± 4.34), with a range from 17 to 36 days (Table 1).

The mean hospitalization period was 32.6 days (25–44 days). During traction, the majority of patients (80%) experienced cervical pain that was addressed with local and oral anti-inflammatory drugs. Pin-site infection was recorded in 6 cases, all of which resolved with local care. Other encountered complications were headache, pin pain, minor neurological symptoms (numbness in the upper limbs that was resolved by subtracting 1 kg off the traction devices), dizziness, and back pain. Most of the patients were mobilized within 48 h after the spinal fusion surgery, and no severe neurological complications, instrumentation failures, or cardiopulmonary complications were observed.

Patients were divided into two groups based on their traction duration: less than 24 days (n = 14) and 24 days or more (n = 14). The mean percentage reduction in primary Cobb angle post-traction was 25.85% (SD = 9.06) for patients with traction duration under 24 days and 26.32% (SD = 10.55) for those with traction duration of 24 days or more.

The mean postoperative reduction for the main curve was 65.3% (SD = 6.3) for the patients that underwent traction for less than 24 days and 63.8% (SD = 6.8) for those with traction duration of 24 days or more.

The Cobb angle of the primary curve, pre-traction, had a mean value of 82.46°, with a minimum value of 68.0° and a maximum of 108.0°. Preoperatively, or post-traction, the Cobb angle of the primary curve was reduced to a mean value of 61.00° (26.09%), ranging from 41.0° to 86.0°. Postoperatively, the primary Cobb angle was further reduced to a mean of 29.54° (64.58% reduction relative to the initial pre-traction Cobb angle), with a minimum of 13.0° and a maximum of 49.0° (Table 2).

The mean pre-traction Cobb angle for the secondary curve was 50.78°, with a range from 30.0° to 100.0°. Following halo-gravity traction, the mean secondary curve angle decreased to 37.22°, with a range of 18.0° to 78.0°, equivalent to a mean percentage reduction of 26.94%. Postoperative measurements showed a further reduction in the secondary Cobb angle to 16.44°, with values spanning from 4.0° to 31.0°. This final correction equated to a mean total percentage reduction of 68.03% from the initial pre-traction angle. The maximum postoperative reduction reached 90%, and the minimum observed was 45.95% (Table 3).

**Figure 2:**
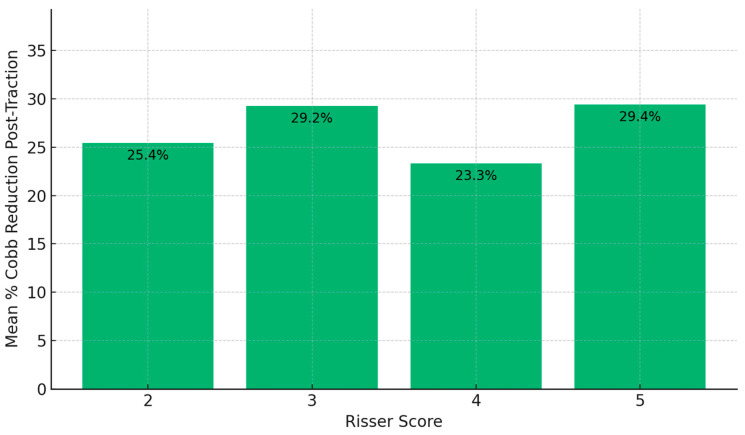
Comparison of mean Cobb angle reduction after traction by Risser score group. Patients with a Risser score of 5 showed the greatest mean reduction, followed by those in Risser groups 3, 2, and 4. Values reflect the percentage decrease in primary curve magnitude following traction.

Analysis by Risser score groups indicated slight differences in Cobb angle reduction. Patients with a Risser score of 5 achieved the highest post-traction mean reduction (29.4%), followed closely by the Risser 3 group (29.24%), compared to Risser 2 (25.44%) and Risser 4 (23.29%) (Figure 2).

**Figure 3:**
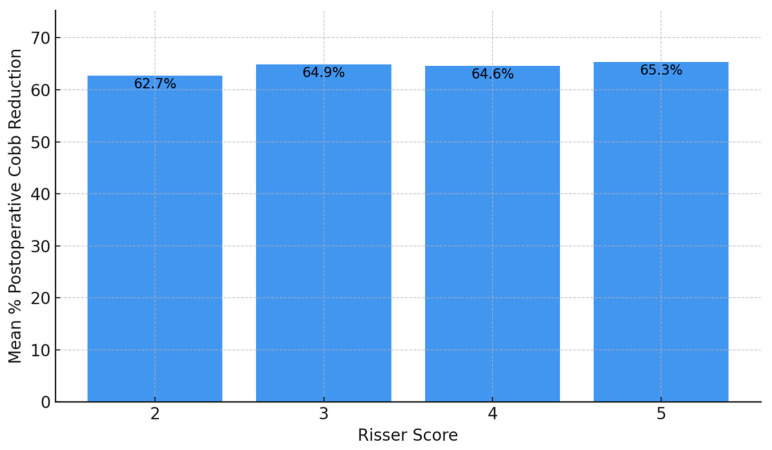
Mean postoperative percentage reduction of the primary scoliotic curve across Risser score groups. Patients with a Risser score of 5 achieved the highest average correction (65.3%), followed closely by Risser 3 (64.87%), Risser 4 (64.63%), and Risser 2 (62.74%), indicating relatively uniform surgical outcomes across different stages of skeletal maturity.

**Figure 4:**
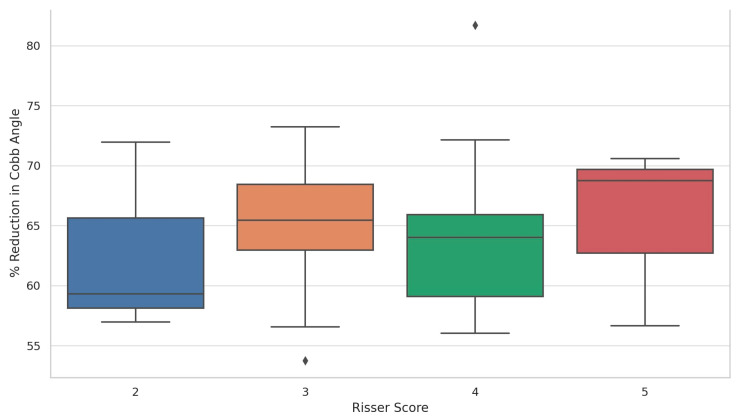
Distribution of postoperative percentage reduction in the primary Cobb angle according to Risser score. The symbols signify the outliers (data points that are located outside the whiskers of the box plot).

Postoperative reduction was relatively uniform: Risser 5 achieved the highest postoperative reduction (65.3%), followed by Risser 3 (64.87%), Risser 4 (64.63%), and Risser 2 (62.74%). When assessing the distribution of postoperative percentage reduction in the primary Cobb angle according to Risser score, patients with a Risser score of 5 demonstrated the highest and most consistent median reduction, approaching 70%, with a narrow interquartile range indicating uniform surgical outcomes. Risser score 3 followed closely, also showing a high median reduction but with a wider spread, suggesting greater variability among patients in this group. Risser scores 2 and 4 showed slightly lower median reductions—approximately 59% and 64%, respectively—with broader interquartile ranges and the presence of outliers, including one above 80% in Risser 4. These findings suggest that substantial postoperative correction was achieved across all stages of skeletal maturity, with no apparent disadvantage for patients with lower Risser scores (Figure 3 and Figure 4).

**Figure 5:**
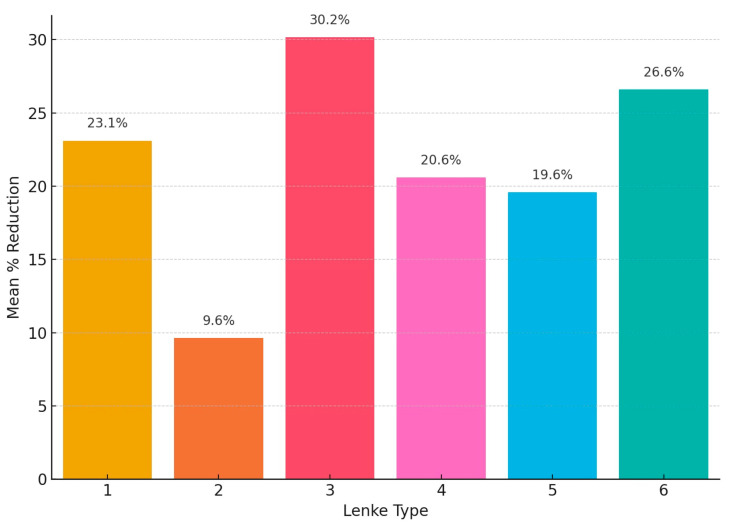
Mean post-traction percentage reduction of the primary scoliotic curve according to Lenke classification. Lenke type 3 curves demonstrated the highest responsiveness to halo traction (30.2%), followed by types 6 (26.6%) and 1 (23.1%). More modest reductions were observed in types 4 (20.6%) and 5 (19.6%), while Lenke type 2 showed the lowest response (9.6%).

The mean post-traction reduction in primary Cobb angle according to Lenke classification varied, and Lenke type 3 curves had the greatest responsiveness to halo traction, with a mean reduction of 30.2%, followed by Lenke type 6 at 26.6% and Lenke type 1 at 23.1%. Lenke types 4 and 5 showed modest reductions of 20.6% and 19.6%, respectively. The lowest response was observed in Lenke type 2, with a mean reduction of only 9.6% (Figure 5).

**Figure 6:**
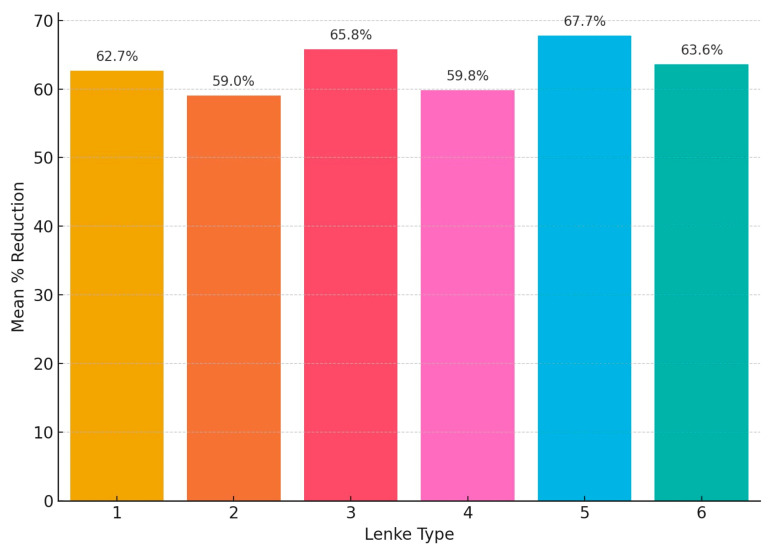
Mean postoperative percentage reduction of the primary scoliotic curve according to Lenke classification. Lenke type 5 demonstrated the greatest surgical correction (67.7%), followed by types 3 (65.8%), 6 (63.6%), and 1 (62.7%). The lowest mean reductions were observed in Lenke types 4 (59.8%) and 2 (59.0%).

Postoperatively, Lenke type 5 demonstrated the highest mean reduction at 67.7%, followed closely by Lenke type 3 with 65.8% and Lenke type 6 with 63.6%. Lenke type 1 achieved a mean reduction of 62.7%, while Lenke types 4 and 2 showed the lowest mean corrections at 59.8% and 59.0%, respectively (Figure 6).

**Figure 7:**
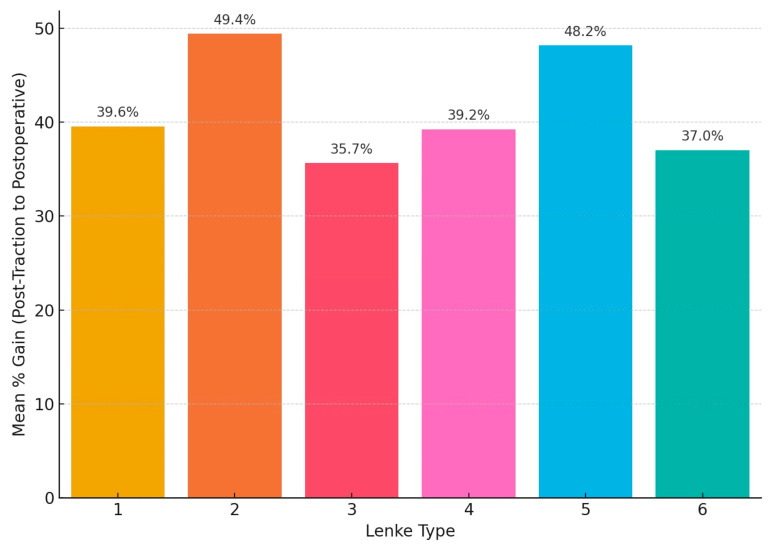
Additional primary curve correction achieved through surgery following preoperative traction, presented by Lenke classification. The greatest surgical gain was observed in Lenke type 2 (49.4%) and type 5 (48.2%), followed by types 1 (39.6%) and 4 (39.2%). Lenke type 6 showed a moderate gain (37.0%), while type 3 demonstrated the lowest surgical gain (35.7%) despite its strong initial response to traction. These results highlight the differential contribution of surgical correction across curve types.

When comparing post-traction to postoperative results, the highest mean surgical gain was observed in Lenke type 2 curves at 49.4%, followed closely by Lenke type 5 at 48.2%. Lenke types 1 and 4 had comparable gains of 39.6% and 39.2%, respectively. Lenke type 3 exhibited the lowest surgical gain at 35.7%, despite having the highest initial correction from traction. Lenke type 6 showed a moderate gain of 37.0%. These values reflect the contribution of surgical intervention in achieving final correction beyond the improvements attained through preoperative traction (Figure 7).

Paired *t*-tests were conducted to evaluate the effectiveness of halo traction and surgical intervention on the primary and secondary Cobb angles. The reduction for the primary curve after traction (T = 14.39, *p* < 0.0001) and the secondary curve after traction (T = 11.67, *p* < 0.0001) was statistically significant. Furthermore, comparisons between pre-traction and postoperative angles demonstrated highly significant reductions in both the primary (T = 53.43, *p* < 0.0001) and secondary curves (T = 14.62, *p* < 0.0001).

Pearson correlation analyses were performed in order to evaluate the relationship between Risser scores, age, and the percentage of Cobb angle reduction. No statistically significant correlations were identified. Risser score showed no meaningful correlation with primary curve post-traction (r = −0.067, *p* = 0.734) or postoperative reduction (r = 0.073, *p* = 0.710). The correlation between age and Cobb angle reduction following halo traction was weakly positive (r = 0.087), with a non-significant *p*-value of 0.659.

Kruskal–Wallis tests were used to evaluate differences in Cobb angle reduction across Lenke types and Risser scores. In our study, there was no statistically significant difference in post-traction primary Cobb angle reduction across Lenke types (H = 8.05, *p* = 0.153) or in postoperative Cobb angle reduction across Risser scores (H = 0.38, *p* = 0.944).

## 4. Discussion

Halo-gravity traction, now a widely adopted preoperative adjunct, enables gradual deformity reduction while patients remain mobile and, importantly, offers a safer pathway to correction with reduced neurologic risk [17].

In the 1960s, Nickel and Perry were pioneers in applying halo skeletal traction at Rancho Los Amigos Hospital to treat cervical paralysis—most often resulting from poliomyelitis—marking a paradigm shift in the management of spinal deformities. Beyond classic halo traction, the halo ring has also been adapted to other skeletal traction systems, including halo-femoral, halo-tibial, and halo-pelvic variants. Building on this concept, in the early 1970s, Pierre Stagnara developed the halo-gravity traction technique, which uses the patient’s body weight to exert gradual corrective forces via the halo device [18,19].

Preoperative halo-gravity traction has been demonstrated in systematic reviews to exhibit a superior safety profile compared to alternative traction techniques, with a notably lower rate of post-traction complications [20]. It has been proven effective in the correction of both the sagittal and coronal deformities, with multiple studies showing radiographic corrections between 25 and 35% [9,21,22,23,24]. Compared to halo-pelvic traction, halo-gravity traction offers several clinical advantages in the management of severe spinal deformities. Halo-gravity traction is less invasive, avoids femoral and pelvic fixation—thereby reducing screw track-related complications—and allows patient mobility throughout treatment, which lowers the risk of bed-related complications such as pressure ulcers and respiratory infections. It is also associated with fewer pin-site infections and less patient discomfort than the more rigid halo-pelvic technique. While halo-pelvic traction may provide greater corrective forces in cases of extremely rigid curves, this benefit is offset by higher morbidity and reduced patient tolerance. Moreover, halo-pelvic traction has been linked to reductions in spinal bone mineral density, with longer traction duration correlating with greater density loss and lower correction rates. These distinctions underscore the clinical preference for HGT, particularly in adolescent idiopathic scoliosis, where safety, mobility, and patient comfort are critical considerations [25,26,27].

In their study, performed on 21 patients with ages ranging from 7 to 20 years, Garabekyan et al. have observed a postoperative correction in the coronal plane of 38%. The patients included in their study underwent halo-gravity traction for a mean period of 77 days with a duration ranging from 22 to 124 days [28]. In our study, after analyzing the radiological data, a correction exceeding 26% in both primary and secondary spinal curves was observed. Compared to the initial Cobb angle, postoperatively a correction of 60% was obtained for the primary and secondary curves.

Currently, there is no consensus regarding the optimal duration of halo-gravity traction (HGT); the goal remains to balance achieving maximal radiographic correction against minimizing traction-related complications. Yang et al. conducted a systematic review including 16 studies and 351 patients, with preoperative traction durations spanning 2 to 13 weeks prior to definitive spinal fusion [29].

Rinella et al. reported a mean traction period of 4 weeks (range: 2–12 weeks) in a retrospective cohort of 33 pediatric patients presenting with severe scoliosis or kyphoscoliosis [30].

A standardized halo-gravity traction protocol was implemented in a pediatric cohort (n = 24; mean age 11.8 years). Traction weight was progressively increased to 50% of each patient’s body weight by the third week and maintained until either neurological warning signs appeared or the 6-week maximum was reached. Most of the correction—approximately 82%—occurred within the initial three weeks, and the mean duration of traction was 42 days [31].

Patients included in our study underwent traction for a mean period of 24.64 days with a maximum of 36 days. The patients that underwent traction for less than 24 days had a mean primary curve correction, preoperatively, of 25.9%, a slight improvement, with 26.3% being observed for the patients that were under traction for more than 24 days.

In our study, no statistically significant correlations were found between Risser score, age, and the percentage of Cobb angle reduction, either after traction or postoperatively. This finding aligns with literature indicating no significant age-related differences in coronal Cobb angle correction following halo-gravity traction [32].

Liu D. et al. have conducted a study on 59 patients in order to compare the efficacy of halo-gravity traction in adolescent and adult patients with severe scoliosis. For the adult patients, they observed a correction of 10.8% after halo-gravity traction and of 52.4% after the spinal fusion surgery. In the adolescent group the angle improved by 19.5% after traction and 57.6% after the surgical intervention [33]. These significant differences were probably achieved mainly due to a wider age span compared to our study.

In our study, Kruskal–Wallis analysis revealed no statistically significant differences in primary Cobb angle reduction following traction across Lenke curve types, nor in postoperative reduction across Risser score groups. These findings suggest that neither curve classification nor skeletal maturity significantly influenced the degree of correction achieved.

Lenke types 3 and 6 demonstrated the highest mean reduction in Cobb angle following traction, while Lenke 2 had the lowest response. Although all patients exhibited substantial improvement following surgical intervention, the degree of postoperative correction differed by curve type, with values ranging from just below 60% (Lenke type 4) to nearly 68% (Lenke type 5). In terms of surgical gain (difference between post-traction and postoperative reductions), Lenke 2 exhibited the most significant operative improvement. Lenke 3 had the lowest surgical gain. Lenke 6 maintained intermediate values across all phases.

One notable strength of our study is that, to the best of our knowledge, it is among the few that specifically examine the relationship between patient age, skeletal maturity as determined by the Risser score, and the effects of halo-gravity traction in treating adolescent idiopathic scoliosis.

The optimal duration of traction is around 24 days, as extending beyond this period offers minimal additional benefits. Factors such as age, Risser score, and curve type do not significantly impact the degree of correction achieved with halo-gravity traction. Our study indicated that within the adolescent age range of 12–17 years, age did not significantly influence the corrective outcome—for both halo traction and surgical treatment—with respect to reduction of the primary Cobb angle. These findings contribute to the growing body of evidence supporting HGT as a valuable tool in the management of complex spinal deformities.

The main drawback of our study would be the retrospective design, as it limits our ability to establish causal relationships between patient characteristics, treatment parameters, and outcomes. Secondly, sagittal plane parameters (thoracic kyphosis and lumbar lordosis) as well as preoperative mobility (bending radiographs) were not consistently documented during or at the end of the traction period. Additionally, our study focused primarily on radiographic outcomes, without detailed assessment of functional improvements, pulmonary function, or quality of life.

Future research should incorporate these patient-centered outcomes to provide a more comprehensive evaluation of treatment success and further investigate the role of preoperative halo-gravity traction and guided growth techniques in the management of early-onset scoliosis, with a focus on their impact on quality of life in patients with adolescent idiopathic scoliosis.

Another limitation of this study is its single-center design and the lack of a control group. Future studies should aim to include larger sample sizes and control groups in order to provide more robust evidence on the effectiveness of halo-gravity traction.

## 5. Conclusions

Halo-gravity traction, followed by surgical correction, offers safe, effective, and significant deformity reduction across adolescent ages and curve types.

A notable reduction in both primary and secondary Cobb angles can be achieved, with an average correction of 60% attained for both curves.

In summary, halo-gravity traction is effective in adolescent idiopathic scoliosis, with optimal results achieved around 24 days. Age, Risser score, and curve type did not significantly influence correction, supporting HGT as a reliable tool regardless of the skeletal maturity of adolescent patients.

## Figures and Tables

**Table 1:** Summary of descriptive statistics for demographic and treatment-related variables in the study group, including patient age, body weight, applied traction weight, and total number of days under traction. Values are presented as mean, standard deviation (SD), minimum, 25th percentile, median, 75th percentile, and maximum.

*Variable*	*Mean*	*SD*	*Min*	*25th %ile*	*Median*	*75th %ile*	*Max*
*Age*	14.86	1.63	12.0	14.0	15.0	16.25	17.0
*Weight*	50.5	7.56	39.0	43.75	51.75	57.25	63.0
*Traction weight*	19.21	2.22	15.0	17.0	20.0	21.0	23.0
*Days of traction*	24.64	4.34	17.0	21.75	24.0	27.0	36.0

**Table 2:** Descriptive statistics for the evolution of the primary scoliotic curve in the study group, including Cobb angle measurements before traction, after traction, and postoperatively, as well as percentage reduction following traction and surgery. Values are presented as mean, standard deviation (SD), minimum, 25th percentile, median, 75th percentile, and maximum.

*Variable*	*Mean*	*SD*	*Min*	*25th %ile*	*Median*	*75th %ile*	*Max*
*Pre-traction Cobb Angle*	82.46	9.47	68.0	76.0	81.5	88.0	108.0
*Post-traction Cobb Angle*	61.0	10.74	41.0	57.5	61.0	64.0	86.0
*Postoperative Cobb Angle*	29.54	7.72	13.0	23.75	29.0	34.0	49.0
*% Reduction Post-traction*	26.09	9.65	9.64	19.97	25.87	31.29	44.16
*% Reduction Postoperative*	64.58	6.47	53.77	59.08	64.39	68.5	81.69

**Table 3:** Descriptive statistics for the evolution of the secondary scoliotic curve in the study group, including Cobb angle measurements before traction, after traction, and postoperatively, as well as percentage reduction following traction and definitive surgical correction. Values are presented as mean, standard deviation (SD), minimum, 25th percentile, median, 75th percentile, and maximum.

*Variable*	*Mean*	*SD*	*Min*	*25th %ile*	*Median*	*75th %ile*	*Max*
*Pre-traction Cobb Angle*	50.78	16.69	30.0	40.5	48.0	56.5	100.0
*Post-traction Cobb Angle*	37.22	13.51	18.0	28.5	34.0	44.5	78.0
*Postoperative Cobb Angle*	16.44	7.52	4.0	11.0	16.0	20.5	31.0
*% Reduction Post-traction*	26.94	9.82	8.89	21.37	26.74	35.35	41.67
*% Reduction Postoperative*	68.03	11.12	45.95	63.5	68.63	74.21	90.0

## Data Availability

The data presented in this study are available on request from the corresponding author due to privacy and ethical reasons.
